# RNA profiling reveals familial aggregation of molecular subtypes in non-BRCA1/2 breast cancer families

**DOI:** 10.1186/1755-8794-7-9

**Published:** 2014-01-31

**Authors:** Martin J Larsen, Mads Thomassen, Qihua Tan, Anne-Vibeke Lænkholm, Martin Bak, Kristina P Sørensen, Mette Klarskov Andersen, Torben A Kruse, Anne-Marie Gerdes

**Affiliations:** 1 Department of Clinical Genetics, Odense University Hospital, Sdr. Boulevard 29, Odense 5000, Denmark; 2 Human Genetics, Clinical Institute, University of Southern Denmark, Odense, Denmark; 3 Epidemiology, Biostatistics and Biodemography, Institute of Public Health, University of Southern Denmark, Odense, Denmark; 4 Slagelse Hospital, Department of Pathology, Slagelse, Denmark; 5 Department of Pathology, Odense University Hospital, Odense, Denmark; 6 Department of Clinical Genetics, Rigshospitalet, Copenhagen University Hospital, Copenhagen, Denmark

**Keywords:** Hereditary breast and ovarian cancer syndrome, RNA profiling, non-BRCA1/2, Gene expression analysis, Microarray analysis, Molecular subtypes, Promoter methylation

## Abstract

**Background:**

In more than 70% of families with a strong history of breast and ovarian cancers, pathogenic mutation in *BRCA1* or *BRCA2* cannot be identified, even though hereditary factors are expected to be involved. It has been proposed that tumors with similar molecular phenotypes also share similar underlying pathophysiological mechanisms. In the current study, the aim was to investigate if global RNA profiling can be used to identify functional subgroups within breast tumors from families tested negative for *BRCA1*/*2* germline mutations and how these subgroupings relate to different breast cancer patients within the same family.

**Methods:**

In the current study we analyzed a collection of 70 frozen breast tumor biopsies from a total of 58 families by global RNA profiling and promoter methylation analysis.

**Results:**

We show that distinct functional subgroupings, similar to the intrinsic molecular breast cancer subtypes, exist among non-*BRCA1*/*2* breast cancers. The distribution of subtypes was markedly different from the distribution found among *BRCA1*/*2* mutation carriers. From 11 breast cancer families, breast tumor biopsies from more than one affected family member were included in the study. Notably, in 8 of these families we found that patients from the same family shared the same tumor subtype, showing a tendency of familial aggregation of tumor subtypes (*p*-value = 1.7e-3). Using our previously developed *BRCA1*/*2*-signatures, we identified 7 non-*BRCA1*/*2* tumors with a *BRCA1*-like molecular phenotype and provide evidence for epigenetic inactivation of *BRCA1* in three of the tumors. In addition, 7 *BRCA2*-like tumors were found.

**Conclusions:**

Our finding indicates involvement of hereditary factors in non-*BRCA1*/*2* breast cancer families in which family members may carry genetic susceptibility not just to breast cancer but to a particular subtype of breast cancer. This is the first study to provide a biological link between breast cancers from family members of high-risk non-*BRCA1*/*2* families in a systematic manner, suggesting that future genetic analysis may benefit from subgrouping families into molecularly homogeneous subtypes in order to search for new high penetrance susceptibility genes.

## Background

Breast cancer is the most common malignant disease and the leading cause of cancer death among women [1]. It is estimated that approximately 5-10% of all breast cancers have a strong hereditary component. High-risk families often show an apparently dominant inheritance pattern of breast cancer cases and are often characterized by early age of onset and presence of ovarian cancers, bilateral breast cancers, and male breast cancers. Germline mutations in *BRCA1* and *BRCA2* only explain the breast cancer risk in approximately one fourth of these families, however, it is expected that additional *BRCA1*/*2* mutations still remain undetected by the screening methods used today [2,3].

Despite intensive research, genetic linkage analysis, genome-wide association studies (GWAS) and most recently next generation sequencing (NGS) exome studies have failed to identify other common high penetrance breast cancer susceptibility genes, like *BRCA1* and *BRCA2*, and it is now generally accepted that no single high penetrance gene is likely to account for a larger fraction of the remaining familial aggregation [4-9]. Instead, the remaining genetic susceptibility is expected to be explained by a mixture of rare high-risk variants and polygenic mechanisms involving more common and/or rare low-penetrance alleles or rare moderate penetrance genes, acting in concert to confer a high breast cancer risk [3,10,11]. Furthermore, a fraction of the familial aggregation of breast cancer cases may be explained by sharing of common environmental risk factors and exogenous hormone use, or be just random aggregation of sporadic breast cancers.

Several rare inactivating mutations in other high penetrance genes have been described to contribute to an increased breast cancer risk, such as *TP53*, *CDH1*, *PTEN*, *STK11*, *RAD51C*, and *RAD51D* and in the low/moderate penetrance genes *ATM*, *CHEK2*, *BRIP1*, and *PALB2* among others (reviewed by Vargas *et al*. [12]). Most are involved in the maintenance of genomic integrity and DNA repair mechanisms and many are associated with multiple cancer syndromes. However, these only explain a minor fraction of the remaining breast cancer risk, leaving the etiology of the remaining high-risk families unexplained. This results in less optimal genetic counseling and risk reducing options for healthy members in these families. It is therefore highly important to identify the cancer predisposition defects of the unsolved high-risk non-*BRCA1*/*2* families.

The histopathologic characteristics of *BRCA1* and *BRCA2* breast tumors are well described. *BRCA1* tumors are frequently grade 3, estrogen receptor (ER) negative, progesterone receptor (PR) negative and HER2-negative (triple-negative), while the majority of *BRCA2* tumors are grade 2/3, ER-positive and HER2-negative [13]. Contrary, familial non-*BRCA1*/*2* breast cancers have been shown to be a very heterogeneous group with varied histopathologic features [14,15].

Gene expression profiling studies of sporadic/unselected breast cancers have revealed the existence of at least five clinically relevant subgroups: basal-like, HER2-enriched, luminal A (lumA) and luminal B (lumB) [16-21]. The molecular subtypes correspond broadly to known histopathological characteristics and are associated with different clinical outcomes. Basal-like cancers are mostly high-grade, triple-negative tumors with high expression of basal epithelial markers such as *CK5*/*14*/*17*; while HER2-enriched cancers are associated with amplification of the *HER2*-gene. LumA cancers are typically low-proliferative, ER + tumors while lumB are high-proliferative, ER + cancers. Luminal cancers show high expression of luminal-associated genes such as *CK8*/*18*. In addition to these four subtypes, a normal-like subtype has also been identified which exhibit high similarity with normal breast epithelium.

Only few gene expression profiling studies of hereditary breast cancers have been published. Recently, we and others have shown that *BRCA1* mostly are basal-like while the majority of *BRCA2* tumors are of luminal subtypes [22-24]. Non-*BRCA1*/*2* cancers have been reported to be distributed across the different molecular subtypes similar to the sporadic cancers; though several studies indicate an enrichment of the lumA subtypes among non-*BRCA1*/*2* tumors compared to sporadic/unselected cases [22,23,25].

Because of the underlying genetic heterogeneity of the non-*BRCA1*/*2* families, the search for more high-susceptibility genes is particular complicated. Even though promising technologies such as exome sequencing provides new opportunities, such attempts have so far failed to identify new high-susceptibility genes [7-9]. The recent negative results using exome sequencing indicate that intelligent stratification is needed in order to reduce heterogeneity. One possible approach to reduce heterogeneity could be to pre-select cases based on molecular tumor characteristics.

In the present study, we have used genome-wide RNA profiling analysis for molecular characterization of familial non-*BRCA1*/*2* tumors. It has been proposed that tumors with similar molecular characteristics are a result of similar pathophysiological mechanisms. Thus, stratifying patients by molecular tumor subgroups based on RNA profiles could potentially facilitate the identification of new breast cancer susceptibility genes by increasing the statistical power of linkage analysis, genome-wide association, or exome sequencing studies. Here, we studied how RNA profiling can be used to identify functional subgroups within tumors from familial non-*BRCA1*/*2* breast cancers and to what extent these subgroupings relate to different patients in the same family. Furthermore, previously developed gene signatures were applied to identify tumors with *BRCA1*-like and *BRCA2*-like phenotypes within the group of non-*BRCA1*/*2* tumors.

## Methods

### Ethics statement

The study was carried out as a retrospective register study and in accordance with the Helsinki Declaration. The study has been approved by The National Committee on Health Research Ethics of Denmark (S-VF-20020142), waiving the requirement for informed consent for the study.

### Patient material

The study was performed on frozen primary breast tumor samples collected from 1982 to 2008. Samples were obtained from the tumor biobanks of Dept. of Pathology, Odense University Hospital and Danish Breast Cancer Cooperative Group (DBCG). Breast tumor tissue from 125 patients with germline mutations in *BRCA1* (*n* = 33) or *BRCA2* (*n* = 22) or with no detectable germline mutation in *BRCA1* or *BRCA2* (*n* = 70) were included in the study. During the period from 2000 to 2005 (64% of the included patients) genetic mutation screening were conducted using a setup consisting of denaturing high-performance liquid chromatography (DHPLC), protein truncation test (PTT) and Sanger sequencing analysis in addition to Multiplex Ligation-dependent Probe Amplification (MLPA) for detection of larger copy number abnormalities. From 2006 (36% of the included patients), the DHPLC method were replaced by Temperature Gradient Capillary Electrophoresis (TGCE) for prescreening of small exons. The patients were all part of a family referred to genetic counseling (Dept. of Clinical Genetics, Odense University Hospital or Dept. of Clinical Genetics, Rigshospitalet) because of a family history of breast cancer. The families were recruited in the period from 2003 to 2009. Inclusion criteria were 1) a pedigree indicating autosomal dominant inheritance of a breast cancer predisposition or 2) presence of ovarian cancer in a pedigree with breast cancer cases or 3) a very young age at diagnosis of breast cancer (before 35 years). A three generation pedigree was constructed on all families depending on the information available from the families. All cancer diagnoses were confirmed by hospital records, histopathology reports or death certificates. Serving as a representative control group, primary breast tumor samples (*n* = 128) were randomly selected among available samples originating from the same department and time period as for the hereditary samples. The family histories of the control patients were unknown, but none of the patients had been referred to genetic counseling at Odense University Hospital, from where the vast majority of patients were recruited, and are therefore here referred to as sporadic. We have previously described gene expression analyses of a subset of the included tumors (*BRCA1*, *BRCA2* and sporadic cases) [24]. Tumor and patient characteristics are summarized in Table 1. Characteristics of the included families are summarized in Table 2.

**Table 1:** Patient and tumor characteristics

		**non**-***BRCA1*/*2 *** **(*n*** **=** **70)**	**BRCA1 (*n*** **=** **33)**	**BRCA2 (*n*** **=** **22)**	**Sporadic (*n*** **=** **128)**
**Estrogen receptor**					
	ER+	8.6%	42.4%	90.9%	83.6%
	ER-	21.4%	57.6%	9.1%	16.4%
**Progesterone receptor**					
	PR+	65.7%	21.2%	72.7%	61.7%
	PR-	34.3%	78.8%	27.3%	38.3%
**HER2 status**					
	HER2+	14.3%	9.1%	4.5%	16.4%
	HER2-	85.7%	90.1%	95.5%	83.6%
**Lymph node**					
	LN+	41.4%	45.5%	63.6%	39.8%
	LN-	52.9%	48.5%	31.8%	58.6%
	NA	5.7%	6.1%	4.5%	1.6%
**Tumor size**					
	Mean tumor size, mm [±SD]	25 [±18]	23 [±10]	25 [±13]	25 [±16]
**Histologic grade**					
	Grade 1	17.1%	9.1%	9.1%	25.0%
	Grade 2	40%	21.2%	50.0%	37.5%
	Grade 3	28.6%	54.5%	31.8%	22.7%
	NA	14.3%	15.2%	9.1%	14.8%
**Tumor type**					
	Invasive ductal carcinoma	78.6%	84.8%	86.4 %	82.0%
	Invasive lobular carcinoma	12.9%	3.0%	9.1 %	9.4%
	Mucinous carcinoma	-	-	-	1.6%
	Medullary carcinoma	1.4 %	6.1%	-	0.8%
	Tubular carcinoma	-	-	-	2.3%
	Metaplastic carcinoma	1.4 %	-	-	-
	Other	-	6.1 %	-	1.6%
	NA	5.7 %	-	4.5%	2.3%
**Age**					
	Median age, years [range]	50 [29–86]	42 [25–74]	43.5 [28–72]	61 [27–95]
	< 50 years	47.1%	63.6%	68.2%	16.4%
	≥ 50 years	52.9%	36.4%	31.8%	83.6%
**Menupause status**					
	Premenopausal	52.9%	60.6%	68.2%	23.4%
	Perimenopausal	2.9%	-	4.5%	11.7%
	Postmenopausal	38.6%	36.4%	22.7%	60.9%
	Other	2.9%	-	-	1.6%
	NA	2.9%	3.0%	4.5%	2.3%

**Table 2:** **Summarized characteristics of the non**-**BRCA1**/**2 families** (**n** = **58**)

	**Percentage**
Breast c. + ovarian c.	36%
2 Breast c.	33%
3 Breast c.	24%
>3 Breast c.	26%
Bilateral breast c.	29%
Early onset breast c. (< 35 years)	10%
Male breast c.	2%
Prostate c.	17%
Colon c.	19%
Cervical c.	14%
Rectal c.	14%
Malignant melanoma	12%
Ventricular c.	12%
Other cancers	52%

### Histopathological review

Samples included in the study contained at least 50% tumor cells determined by representative hematoxylin-eosin stainings. Tumor samples were macro-dissected to increase purity and remove normal cell-enriched areas. Histopathological data and ER, PR, and HER2 status determined by immunohistochemistry (IHC) were obtained from DBCG. In addition, gene-expression data were used to determine ER, PR, and HER2 status as previously described [24].

### Gene expression analysis

Gene expression analysis was performed using a customized version of Agilent SurePrint G3 Human GE 8x60K Microarray and raw data were pre-processed as previously described [24]. Microarray data have been deposited to the Gene Expression Omnibus (GSE49481).

### Detection of promoter methylation

Hypermethylation of the *BRCA1* and *BRCA2* promoter regions were determined by the methylation-specific MLPA kit ME001C (MRC-Holland) using DNA extracted from freshly frozen tumor tissue. Blood DNA from 6 healthy donors were used as reference. CpGenome Universal Methylated DNA (Millipore) was used as positive control. A methylation ratio > 0.2 was considered as positive promoter methylation. Methylation status was successfully determined in 235 out of the 253 samples analyzed by gene expression analysis.

### Data analysis

#### Unsupervised methods

Unsupervised hierarchical clustering (Euclidian metric, complete linkage) and principal-component analysis (PCA) were carried out in Qlucore Omics Explorer. Expression levels of each gene had been standardized to zero mean and unit variance.

#### Molecular subtype classification

PAM50 subtype classifier described by Parker *et al*. was used to classify tumors into five intrinsic molecular subtypes using the R package *genefu*[20]. Probes for all 50 PAM50 genes were present on the Agilent microarray used.

#### Familial aggregation testing

Aggregation of breast cancer subtypes in the observed families was tested using computer simulation. In the simulation, the observed tumor samples were randomly assigned to each family with the number of assigned tumors as observed in the family. The counts for tumor subtypes falling into each family were recorded. The procedure was repeated for 100,000 times based on which the empirical *p*-value for testing familial aggregation of a tumor subtype was calculated as the proportion of replicates with eight or more families having two or more tumors of the same subtype.

A microarray gene expression dataset published by Hedenfalk *et al*. was used for confirmative assessment of familial aggregation of molecular breast cancer subtypes [26]. Pre-processing steps of the dataset are described in the supplementary information (see Additional file 1: Methods S1).

#### Prediction of BRCA1 and BRCA2 associated breast cancers

Two subtype specific gene signatures have previously been developed by our group using the included *BRCA1*, *BRCA2* and sporadic samples [24]. Using the 110-gene basal *BRCA1* signature, we were able to distinguish *BRCA1* from sporadic tumors among basal-like samples and the 100-gene lumB *BRCA2* signature enabled us to separate *BRCA2* tumors from sporadic among lumB samples, both with high accuracies. Here, we used the gene signatures, employing the support vector machines algorithm (linear kernel), to identify tumors with *BRCA1*-like and *BRCA2*-like phenotype within basal-like and lumB non-*BRCA1*/*2* tumors, respectively.

## Results

### Pathological characteristics of patient material

In the present study, frozen primary breast tumor samples were collected from 70 non-*BRCA1*/*2*, 33 *BRCA1*, 22 *BRCA2*, and 128 sporadic cases. Median age of diagnosis was 50 years for the non-*BRCA1*/*2* patients compared to 42 years (*BRCA1*) and 43.5 years (*BRCA2*) for mutation carriers and 61 years for sporadic cancer patients. Tumor and patient characteristics are summarized in Table 1. The majority of *BRCA1* tumors were ER- (58%) and tumor grade 3, while *BRCA2* tumors were predominantly ER + (91%) and grade 2 or 3. Tumor grades of non-*BRCA1*/*2* tumors were distributed more evenly, but compared to sporadic tumors, overall slightly higher. Of the non-*BRCA1*/*2* tumors, 17% displayed triple-negative phenotype (ER-/PR-/HER2-), compared to 8% of sporadic tumors, 55% of *BRCA1* tumors, and 9% of *BRCA2* tumors.

### Subgroups within non-BRCA1/2 tumor samples are predominantly determined by ER status and molecular subtypes

**Figure 1:**
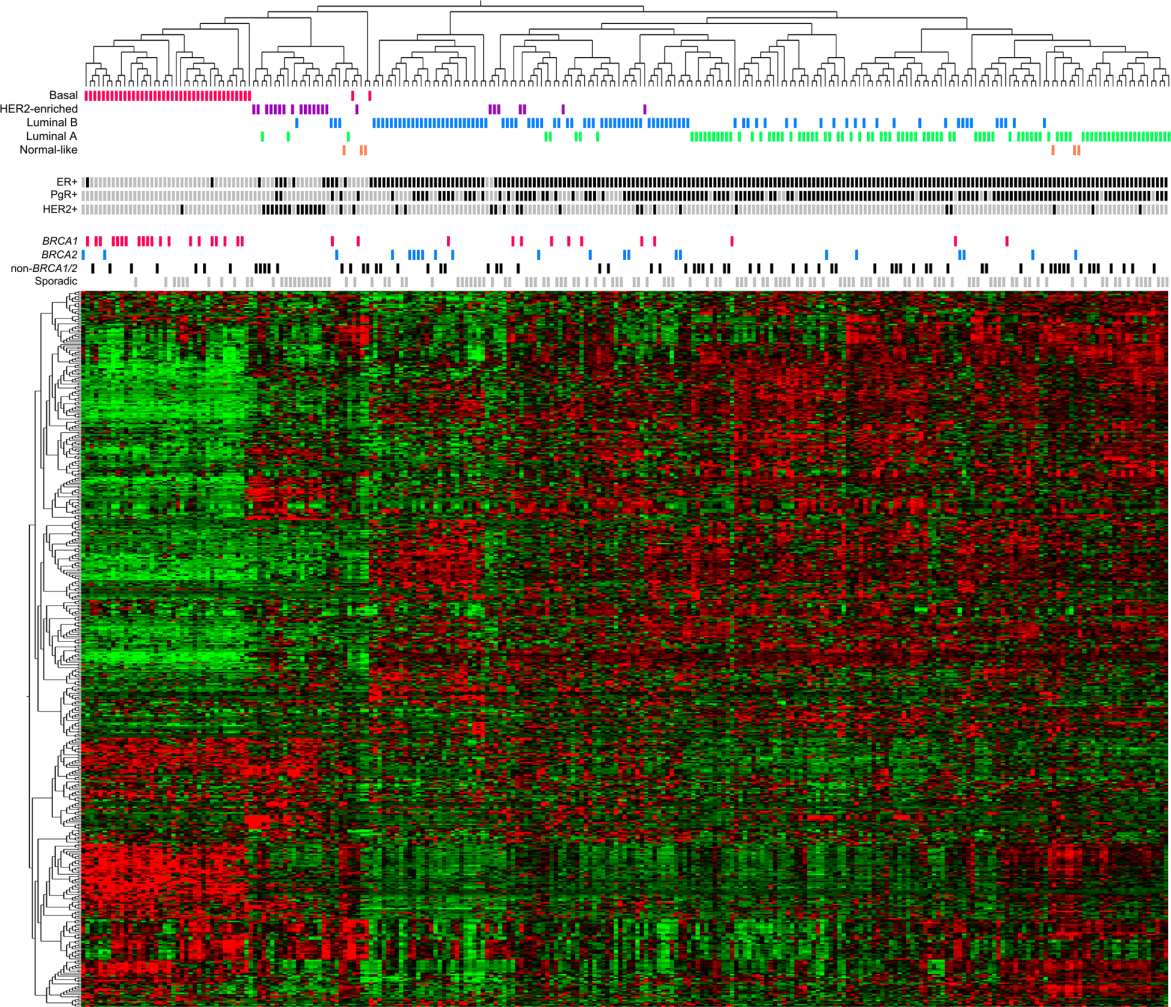
**Hierarchical clustering of 253 breast tumor samples using the 500 most variant genes across all samples.** In the heat map rows correspond to genes and columns to samples. Red indicates elevated expression, green reduced expression. Status bars designate molecular subtype, hormone receptor status and sample group.

Genome wide gene expression analyses resulted in a data set consisting of 22,171 probes with unique gene symbols assigned. We performed unsupervised two-dimensional hierarchical clustering analysis of the whole series of tumors available (70 non-*BRCA1*/*2*, 33 *BRCA1*, 22 *BRCA2* and 128 sporadic) using the 500 most variant genes (Figure 1). Two main branches were formed, separating ER- samples from ER + samples. The ER- cluster could be further subdivided into a triple-negative cluster, covering the majority of the *BRCA1* tumors, a HER2+/ER-/PR- cluster and a smaller mixed cluster. Tumors were classified into the intrinsic molecular subtypes (basal-like, lumA, lumB, HER2-enriched, or normal-like) by the PAM50 classifier developed by Parker *et al*. [20]. The triple-negative cluster consisted of only basal-like tumors, while the majority of HER2-enriched tumors were found in the HER2+/ER-/PR- cluster. Within the ER + main cluster, sub-branches enriched for lumA and lumB samples were present. 13/22 *BRCA2* and 8/12 ER + *BRCA1*-associated tumors grouped in the lumB branches. Non-*BRCA1*/*2* and sporadic tumors were not confined to any specific subcluster.

Clearly, hormone receptor status and molecular subtypes were the major determinants of the structure of the hierarchical clustering when including the whole series of samples. To clarify to what extent clustering of non-*BRCA1*/*2* tumors were influenced by the presence of the *BRCA1*, *BRCA2* and sporadic tumors, hierarchical clustering analysis was conducted using only data from non-*BRCA1*/*2* tumor samples (Additional file 2: Figure S1). The clustering pattern of non-*BRCA1*/*2* was found to be comparable to the initial clustering analysis, again predominantly associated with hormone receptor status and molecular subtypes.

**Figure 2:**
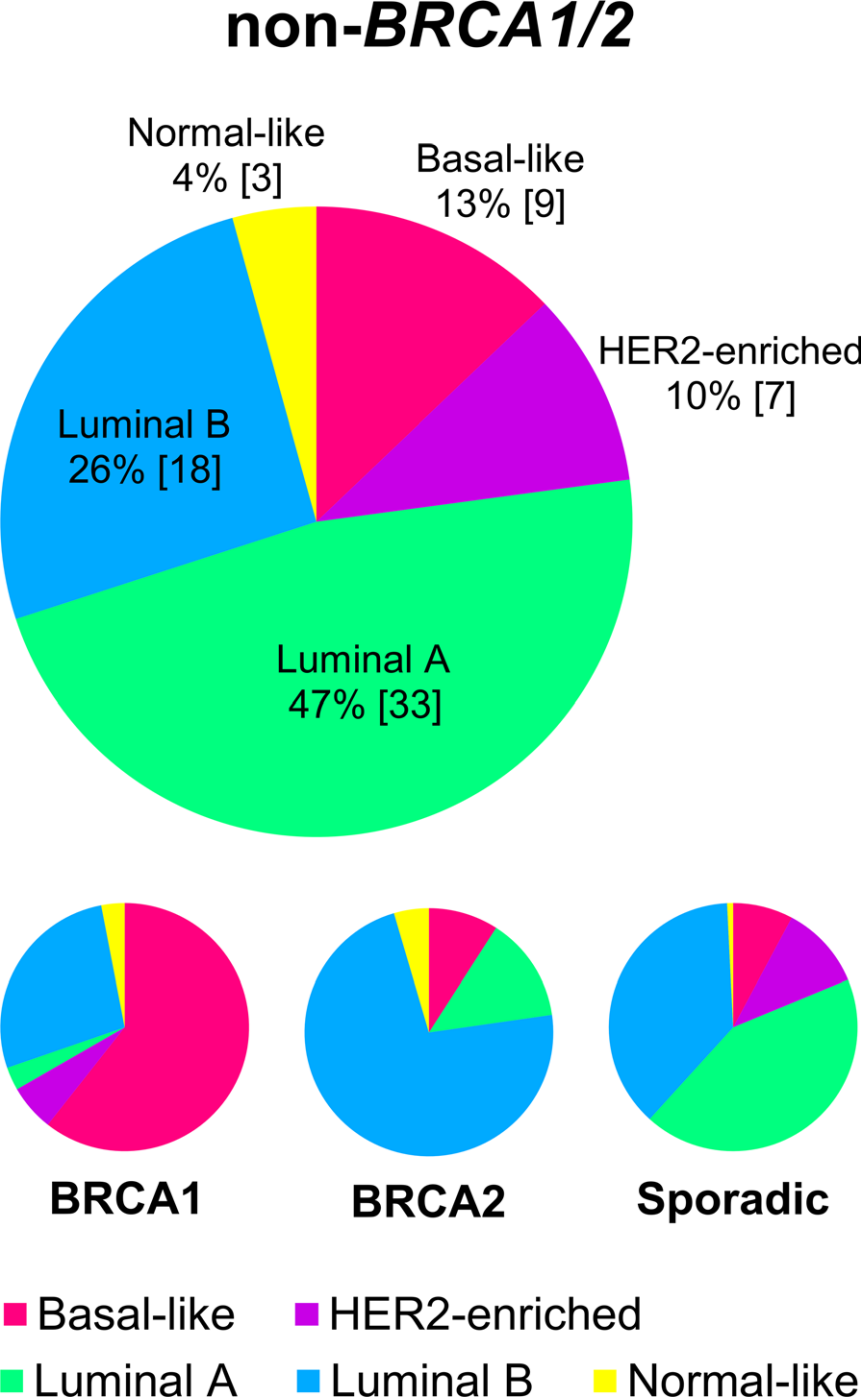
**Association between hereditary breast cancers and molecular subtypes.** Distribution of molecular subtypes among familial non-*BRCA1*/*2*, *BRCA1*, *BRCA2* and sporadic breast cancer samples. Tumors were classified into molecular subtypes using the PAM50 classifier. The molecular subtypes of the *BRCA1*, *BRCA2* and sporadic breast cancer samples have been described previously in Larsen *et al*. [24]. Numbers in brackets refer to number of samples in each group.

The distribution of the PAM50 classified intrinsic molecular subtypes within non-*BRCA1*/*2*, *BRCA1*, *BRCA2* and sporadic tumors was determined (Figure 2, Additional file 3: Figure S2, Additional file 4: Table S1). All five molecular subtypes were found within the non-*BRCA1*/*2* tumor class. The majority of non-*BRCA1*/*2* tumors were mainly classified as lumA (33/70, 47%) or lumB (18/70, 26%), while 9 tumors were basal-like (13%), 7 HER2-enriched (10%) and 3 were normal-like (4%). The distribution of molecular subtypes among the non-*BRCA1*/*2* tumors was similar to the distribution found in the sporadic tumors; although a tendency towards more non-*BRCA1*/*2* tumors were basal-like while fewer were classified as lumB. On the contrary, non-*BRCA1*/*2* tumors were markedly different from both *BRCA1* and *BRCA2* tumors which were highly associated with the basal-like subtype (p = 1.2 × 10^-6^, Fisher’s exact test) and lumB subtype (p = 1.2 × 10^-4^, Fisher’s exact test), respectively.

### BRCA-like tumors can be identified among non-*BRCA1*/*2* tumors

**Figure 3:**
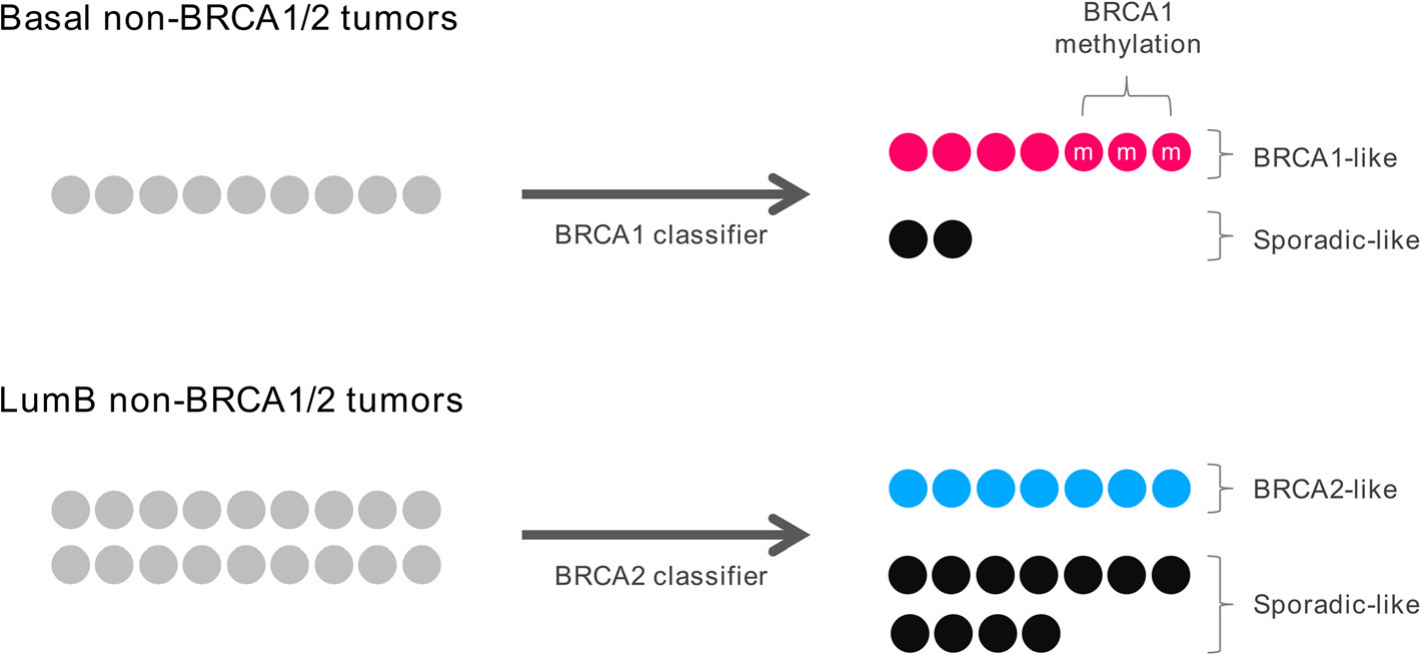
**Prediction of *BRCA1 *and *BRCA2 *association among familial non**-***BRCA1***/***2 *tumors.** Subtype specific gene signatures were applied to predict *BRCA1* and *BRCA2* association among basal-like and lumB non-*BRCA1*/*2* tumors, respectively.

We have previously developed two subtype specific gene signatures, a 110-gene basal *BRCA1* signature and a 100-gene lumB *BRCA2* signature [24]. These signatures were here used to predict *BRCA1* and *BRCA2* association among basal-like and lumB non-*BRCA1*/*2* tumors, respectively (Figure 3). Seven basal-like tumors out of nine were classified as *BRCA1*-like (78%) and the remaining 2 as sporadic-like (22%). Among the lumB subtype, 7 samples were classified as *BRCA2*-like (39%) and 11 as sporadic-like (61%). Detailed prediction results can be found in Additional file 5: Table S2.

### Epigenetic inactivation of BRCA1 by promoter methylation

To identify aberrant methylation patterns in *BRCA1* and *BRCA2* promoter regions, tumor tissues were analyzed using the ME001C methylation-specific MLPA kit (MRC-Holland). Promoter methylation patterns were obtained from all 33 *BRCA1* tumors, 18 out of 22 *BRCA2* tumors, 64 out of 70 non-*BRCA1*/*2* tumors and 120 out of 128 sporadic tumors. Six samples were found to be positive for methylation of the *BRCA1* promoter, counting three basal-like non-*BRCA1*/*2* tumors, one normal-like non-*BRCA1*/*2* tumor, one basal-like sporadic tumor and one lumB sporadic tumor. Notably, the three basal non-*BRCA1*/*2* tumors classified as *BRCA1*-like were all found to have BRCA1 promoter hypermethylation. None of the cases showed methylation of the *BRCA2* promoter.

Methylation of the *BRCA1* promoter was associated with a significant reduction of the *BRCA1* mRNA expression level compared to tumors not methylated in the *BRCA1* promoter (*p* = 0.024, *t*-test, Additional file 6: Figure S3). Notably, four out of the 6 methylated samples exhibited extremely low *BRCA1* expression compared to the remaining non-methylated samples.

### Molecular subtypes aggregates within non-*BRCA1*/*2* families

**Figure 4:**
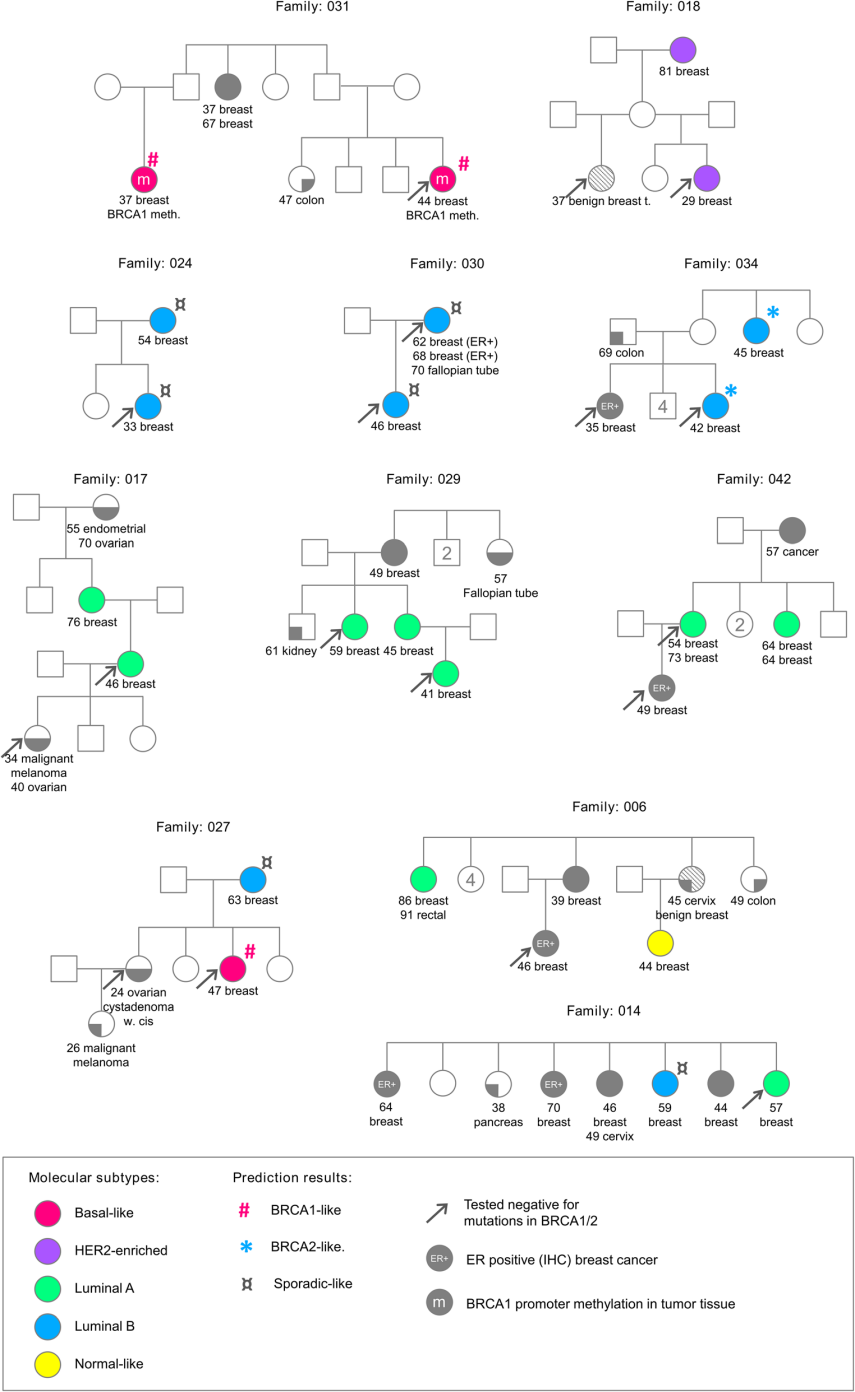
**Pedigrees of 11 selected non**-***BRCA1***/***2 *families included in this study.** Molecular subtypes determined by the PAM50 classifier and positive *BRCA1* promoter methylation are visualized. For basal-like and lumB tumors, the result of the prediction of *BRCA1* and *BRCA2* association are given. If available, IHC ER status is indicated from family members not analyzed in this study.

The 70 non-*BRCA1*/*2* patients included in the study originated from 58 breast and/or ovarian cancer families tested negative for germline mutations in *BRCA1* or *BRCA2*. From 10 of these families, breast tumor biopsies were available from two affected family members and from a single family we obtained tumor material from three patients. The pedigrees of these eleven families are shown in Figure 4, along with corresponding molecular subtypes, *BRCA1* methylation status and prediction results obtained by the basal *BRCA1*-classifier and the lumB *BRCA2*-classifer. In 8 of the families we found that affected members of the same family shared the same tumor subtype. Three families were characterized by only lumA tumors, 3 families had only lumB tumors, 1 family contained only HER2-enriched tumors, and 1 family only basal-like tumors (Table 3).

**Table 3:** Subtype classification of tumors from 11 families with tumor material from more than one affected individual

**FamilyID**	**Basal-like**	**HER2-enriched**	**Luminal A**	**Luminal B**	**Normal-like**
**031**	2	-	-	-	-
**018**	-	2	-	-	-
**017**	-	-	2	-	-
**029**	-	-	3	-	-
**042**	-	-	2	-	-
**024**	-	-	-	2	-
**030**	-	-	-	2	-
**034**	-	-	-	2	-
**027**	1	-	-	1	-
**006**	-	-	1	-	1
**014**	-	-	1	1	-

To evaluate the significance of this finding, we performed familial aggregation testing using computer simulation as described in Materials and Methods. The result of the simulation study confirmed that the degree of familial subtypes aggregation was significant, non-random aggregation (*p*-value = 1.7e-3).

In order to confirm the observed tendency in independent samples, we used a dataset published by Hedenfalk *et al*. [26]. This dataset contained gene expression profiles for tumors of 16 non-*BRCA1*/*2* individuals, belonging to 8 different families. From five families, two or three tumors were analyzed, in total 12 samples. Subtype prediction was carried out by the PAM50 classifier (Table 4). Even though only 19 of the 50 probes were present in the Hedenfalk dataset, the same tendency was observed here. In 3 two-case families, members from the same family shared the same tumor subtypes, whereas in one family 2 normal-like tumors and one basal-like tumor were found, and another family had three different subtypes. Simulation analysis confirmed that the degree of familial aggregation was significant (*p*-value = 0.017).

**Table 4:** Subtype classification of tumors from Hedenfalk et al

**FamilyID**	**Basal-like**	**HER2-enriched**	**Luminal A**	**Luminal B**	**Normal-like**
**L101**	2	-	-	-	-
**L414**	-	-	2	-	-
**L505**	-	-	-	2	-
**L99**	1	-	-	-	2
**L16**	1	1	1	-	-

## Discussion

In this report, we investigated genome-wide RNA profiles of tumors from familial breast cancer cases where no *BRCA1* or *BRCA2* mutations could be identified by traditional genetic analysis for germline mutations. The search for high-susceptibility genes that can explain the breast cancer risk in the remaining non-*BRCA1*/*2* families is complicated by their underlying genetic heterogeneity, and a probable explanation of the low success rate of attempts to identify new high-risk alleles. Obviously, intelligent stratification of cases is needed in order to reduce this heterogeneity. The aim of this study was to explore if RNA profiling can be used to identify homogeneous subgroups among these families. Subgrouping into homogeneous subsets could be of considerable value for further genetic analysis and may facilitate the identification of new high penetrance breast cancer susceptibility genes. Here we describe RNA based subgroupings among non-*BRCA1*/*2* breast tumors, displaying strong associations with the previously described intrinsic molecular subtypes. Within these subgroups we identified tumors with *BRCA1*-like and *BRCA2*-like molecular phenotype. Furthermore, we showed that breast cancer family members from the same family often show identical molecular subtypes.

### Molecular subtypes of familial non-*BRCA1*/*2* tumors

Unsupervised hierarchical clustering was used to reveal overall patterns and for identification of subgroups within the familial non-*BRCA1*/*2* tumors. The clustering displayed a high level of heterogeneity with sub-branches mainly related to hormone receptor status and the intrinsic molecular tumor subtypes. Comparable subgroupings were observed when including a set of 33 *BRCA1*, 22 *BRCA2* and 128 sporadic tumors from our previous study [24]. Here, familial non-*BRCA1*/*2* tumors clustered with tumors from sporadic and with *BRCA* germline mutation carriers, indicating that the subgroups of the familial tumors are very similar to the intrinsic molecular subtypes found among sporadic/unselected breast cancers [16,17]. Similar observations have been described by others using gene expression analysis and immunohistochemical surrogate markers for subtype classification [15,25,27-29].

In our study, familial non-*BRCA1*/*2* tumors classified predominantly as luminal subtype with lumA tumors accounting for almost half of the tumors. The distribution of subtypes was very similar to the distribution found among sporadic tumors. Only few studies have investigated molecular subtypes with relation to familial non-*BRCA1*/*2* tumors [22,23,25]. Jönsson *et al*. and Waddell *et al*. reported that lumA tumors were the most predominant luminal subtype among familial non-*BRCA1*/*2* tumors, similar to what we observed in our study group, while Nagel *et al*. reported that lumB tumors were most frequent. These variations were however minor and might be due to differences in the inclusions criteria and subtyping methodologies used.

### Identification of BRCA-like tumors among familial non-*BRCA1*/*2* tumors

*BRCA1* and *BRCA2* mutation screening technology traditionally include Sanger sequencing in combination with MLPA analysis. However, a fraction of mutations will still not be detected. We used a basal *BRCA1* signature from our previous study to predict whether tumors with a *BRCA1*-like molecular phenotype were present among basal non-*BRCA1*/*2* samples [24]. We found that 7/9 (78%) basal non-*BRCA1*/*2* samples were *BRCA1*-like. In a similar approach using our lumB *BRCA2* signature, we identified 7/18 (39%) lumB non-*BRCA1*/*2* tumors to be *BRCA2*-like. This could indicate a *BRCA1*/*2*-deficiency in these tumors, either caused by an inactivating mutation not detected by current technology or epigenetic silencing such as promoter hypermethylation of the *BRCA1*/*2* genes or other susceptibility genes in the same pathway.

A similar approach was used to identify *BRCA1*-like and *BRCA2*-like tumors among non-*BRCA1*/*2* tumors in a study by Joosse *et al*. using array-CGH profiles. In one study, two out of 48 non-*BRCA1*/*2* breast tumors exhibited chromosomal aberration patterns similar to those found in *BRCA1*-mutated tumors, of which hypermethylation of the *BRCA1* promoter was demonstrated in one sample [30]. In another study focusing on *BRCA2*, 12 tumors out of 89 tumors were found to exhibit a high level of similarity with *BRCA2*-mutated breast tumors [31]. In two cases, additional indications of a dysfunctional *BRCA2* gene function were observed.

### Promoter methylation

To find further evidence for *BRCA1* or *BRCA2* involvement in the familial non-*BRCA1*/*2* tumors, we performed methylation-specific MLPA analysis of the *BRCA1* and *BRCA2* promoter regions. Four non-*BRCA1*/*2* samples were found to be methylated in the *BRCA1* promoter region (3 basal-like, 1 normal-like). Notably, the three basal-like tumors were all found also to have a *BRCA1*-like expression profile. This supports the hypothesis that tumors with epigenetic silencing of *BRCA1* by promoter hyper-methylation are similar to *BRCA1* mutated tumors [30,32-34]. The absence of *BRCA2* promoter methylation in our sample group is in line with previous observations [35].

### Aggregation of molecular subtypes within non-*BRCA1*/*2* families

It is well established that families carrying a germline *BRCA1* mutation have increased incidence of basal-like/triple-negative breast cancers, while germline *BRCA2* mutations predispose to cancers of the lumB subtype [22-24]. If the same tendency is true for defects in other high penetrance breast cancer genes, tumors from genetically related patients would exhibit related molecular subtypes, as it is the case for *BRCA1 and BRCA2* families. The 70 familial non-*BRCA1*/*2* cases included in the current study, originated from 58 breast cancer families with tumor material from one affected family member and from 11 families with tumor material from more than one affected individual. This gave us a unique opportunity to investigate the “*same gene* – *same subtype*” hypothesis. Convincingly, we found that members of the same family shared the same tumor subtype in 8 of the 11 families. Three of the families were characterized by lumA tumors only (including the 3-case family), three families had lumB tumors, one had HER2-enriched tumors and one had only basal-like tumors. By computer simulation we found that the observed familial aggregation was highly unlikely to be a chance observation.

In order to confirm our observations, we identified a dataset from a study by Hedenfalk *et al*. where gene expression data were available from tumors from 5 high-risk families negative for BRCA1/2 mutations [26]. Three 2-case families showed full concordance, while a 3-case family contained 2 normal-like samples and 1 basal-like, and another 3-case family was represented by 3 different subtypes. Again, computer simulation confirmed this as significantly different from random.

Our observations indicate an underlying common genetic basis in these families and that the cancers in the families are unlikely to be sporadic of origin. The family members may carry an inherited susceptibility not just to breast cancer but to a particular subtype of breast cancer. In support of the “*same gene* – *same subtype*” hypothesis, in a study by Waddell *et al*. the authors noticed that all tumor biopsies from *ATM* mutation carriers included in their study were classified as luminal (4 lumB and 2 lumA) [36]. Within a large non-*BRCA1*/*2* family, four out of five family members were classified as lumA. Furthermore, a study by Nagel *et al*. included a group of 26 breast tumors from CHEK2 1100delC carriers; all were classified as luminal tumors (8 LumA and 18 LumB). On the contrary, a study analyzing tumors using IHC noted that different tumors within the same family frequently belonged to different phenotype categories [37]. This discrepancy may, however, be related to differences in methodology and/or inclusion criteria. In addition, in a recent study by Didraga et al., a specific tumor arrayCGH profile has been shown to cluster within a subgroup of non-BRCA1/2 families [38].

Segregation studies have indicated that the remaining genetic susceptibility within non-BRCA1/2 families may be explained by a mixture of rare high-risk variants and polygenic mechanisms involving more common and/or rare low-penetrance alleles or rare moderate penetrance genes, acting in concert to confer a high breast cancer risk. Our observations that family members often shares the same molecular subtype may be compatible with both scenarios. The cancer-risk and tumor subtype may be a result of either private mutations in high-penetrance genes or be a result of multiple low/moderate penetrant genes acting in concert.

In our study, *BRCA1* promoter methylation was detected in breast tumors from both women in a family with basal-like tumors (Family 031). Involvement of *BRCA1* was further supported as both tumors were predicted to be *BRCA1*-like by expression analysis. To investigate if *BRCA1* methylation could be due to an inherited trait in this family we analyzed DNA from peripheral blood leukocytes available from one of the women. No aberrant *BRCA1* methylation pattern in the blood sample was detected by the MLPA method.

In the 3 pure lumB families, we found full concordance in the *BRCA2*-like prediction results between family members of the same family. Tumors from one lumB family were classified as *BRCA2*-like, which could indicate inactivation of *BRCA2* or related genes. In two other lumB families all members were classified as non-*BRCA2*-like (sporadic-like), indicating that *BRCA2* is less likely to be involved in the development of cancer in these families.

## Conclusions

Familial non-*BRCA1*/*2* breast tumors comprising a molecularly heterogeneous group of cancers can be further classified by RNA profiling into subgroups showing high resemblance to the intrinsic molecular subtypes. To the best of our knowledge this is the first study to systematically demonstrate that members of the same family often share the same molecular breast cancer subtype, indicating that germline inactivation of certain genes may give rise to specific breast cancer subtypes. These findings could be highly relevant when analyzing data from next generation sequencing of affected family members. Although additional validation studies are required to confirm this tendency, our findings suggest that future genetic analysis may benefit from stratifying tumors/families into molecularly homogeneous subtypes in order to identify new high penetrance susceptibility genes.

## Competing interests

The authors have declared that no competing interests exist.

## Authors’ contributions

MJL, AMG, MT, and TAK conceived the study and designed the experiments; AVL, MB, MKA and AMG were involved in acquisition of materials and data; MJL and KPS carried out the experiments; MJL performed the data analysis; MJL, AMG, MT, QT, and TAK were involved in the interpretation of the results; MJL, with the support of all authors, wrote the paper. All authors were involved in revising the paper and had final approval of the submitted version.

## Pre-publication history

The pre-publication history for this paper can be accessed here:

http://www.biomedcentral.com/1755-8794/7/9/prepub

## Supplementary Material

Additional file 1: **Methods S1.** Preparation of validation dataset. Click here for file

Additional file 2: Figure S1. Unsupervised hierarchical clustering of non-BRCA1/2 tumor samples using the 200 most variant genes. Click here for file

Additional file 3: Figure S2. PCA plots visualizing molecular subtype classifications. Click here for file

Additional file 4: Table S1. Distribution of predicted molecular subtypes within *BRCA1*, *BRCA2*, non-*BRCA1*/*2* and sporadic tumors using PAM50 signature. Click here for file

Additional file 5: Table S2. Patients characteristics and *BRCA1*-like and *BRCA2*-like predictions results for familial non-*BRCA1*/*2* patients. Click here for file

Additional file 6: Figure S3. Relations between *BRCA1* gene expression and *BRCA1* promoter methylation and PAM50 molecular subtypes. Click here for file
