# Supplementary material for: RNA profiling reveals familial aggregation of molecular subtypes in non-BRCA1/2 breast cancer families
Source: BMC Med Genomics. 2014 Jan 31;7:9. doi: 10.1186/1755-8794-7-9 (PMC3909442; doi:10.1186/1755-8794-7-9)
Supplement: Additional file 1: — Methods S1. Preparation of validation dataset. [file 1755-8794-7-9-S1.pdf]

Methods S1: Preparation of validation dataset

A microarray gene expression dataset published by Hedenfalk *et al.* was used for confirmative assessment of familial aggregation of molecular breast cancer subtypes [1]. Files were available as supporting material (http://www.pnas.org/content/100/5/2532/suppl/DC1). The dataset contained gene expression data from 16 non-*BRCA1/2* tumors (15 primary tumors and one metastatic samples) originating from 8 families. Only primary tumor samples were included in the following analysis. SOURCE webtool (http://source.stanford.edu) was used to map the available CloneID annotated probes to gene symbols. In cases of multiple probes per gene symbol only the probe with the highest variance across all samples was kept before $\log_2$ transforming the data.

Classification of the samples into molecular subtypes was performed using the PAM50 classifier as described in the materials and methods section. 19 out of the 50 genes comprising PAM50 could be mapped to a probe in the Hedenfalk dataset.

1. Hedenfalk I, Ringnér M, Ben-Dor A, Yakhini Z, Chen Y, Chebil G, et al. Molecular classification of familial non-BRCA1/BRCA2 breast cancer. Proc Natl Acad Sci U S A. 2003 Mar 4;100(5):2532–7.
