# Supplementary material for: RNA profiling reveals familial aggregation of molecular subtypes in non-BRCA1/2 breast cancer families
Source: BMC Med Genomics. 2014 Jan 31;7:9. doi: 10.1186/1755-8794-7-9 (PMC3909442; doi:10.1186/1755-8794-7-9)

Figure S1. Unsupervised hierarchical clustering of non-*BRCA1/2* tumor samples using the 200 most variant genes.

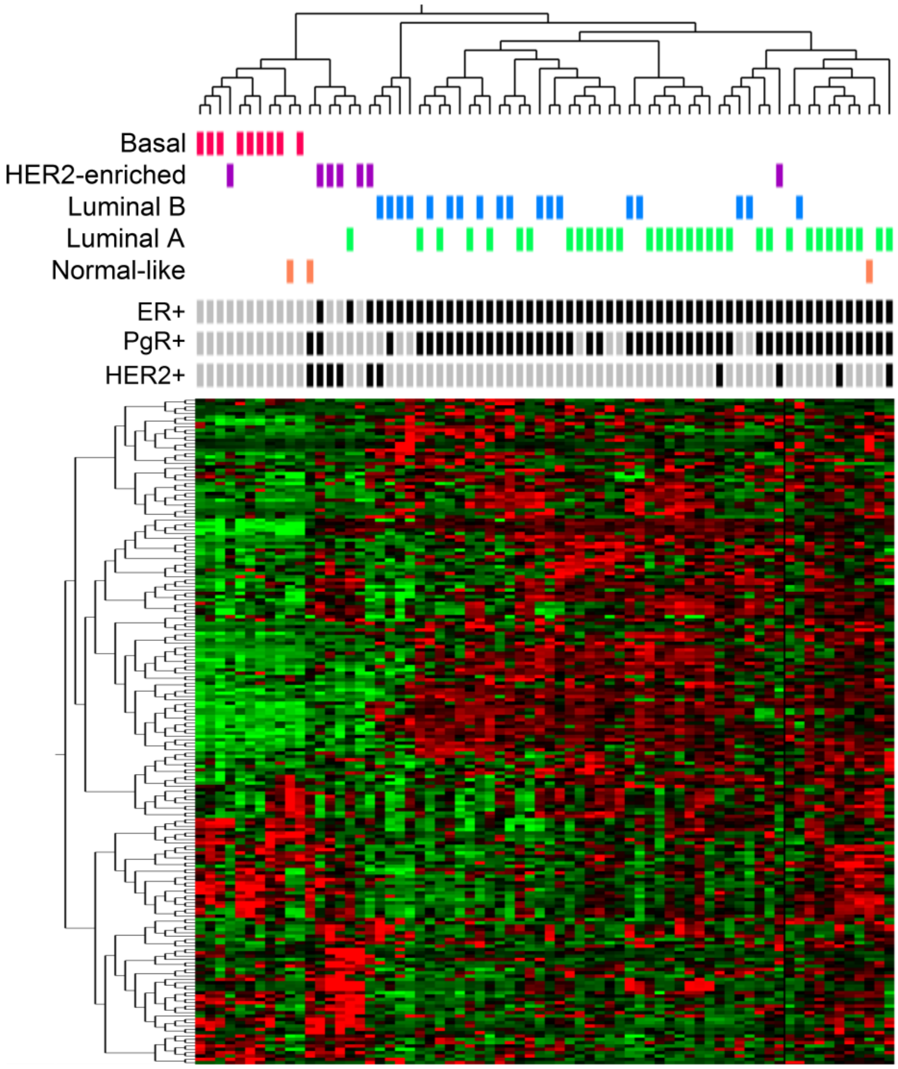

Supplement: Additional file 2: Figure S1. — Unsupervised hierarchical clustering of non-BRCA1/2 tumor samples using the 200 most variant genes. [file 1755-8794-7-9-S2.pdf]
