# Supplementary material for: RNA profiling reveals familial aggregation of molecular subtypes in non-BRCA1/2 breast cancer families
Source: BMC Med Genomics. 2014 Jan 31;7:9. doi: 10.1186/1755-8794-7-9 (PMC3909442; doi:10.1186/1755-8794-7-9)

Figure S2. PCA plots visualizing molecular subtype classifications. PCA plots show 253 breast tumor samples plus the 5 PAM50 centroids defined by Parker *et al.*, 2009. The 50 genes comprising PAM50 were used to construct the PCA plots. A: Black dots represent breast tumor samples and colored dots represent the PAM50 centroids. B: Colored dots represent the molecular subtype of all 253 samples and white dots are the PAM50 centroids. C-F: The molecular subtypes are highlighted for sporadic samples, non-BRCA1/2 samples, BRCA1 samples, and BRCA2 samples, respectively.

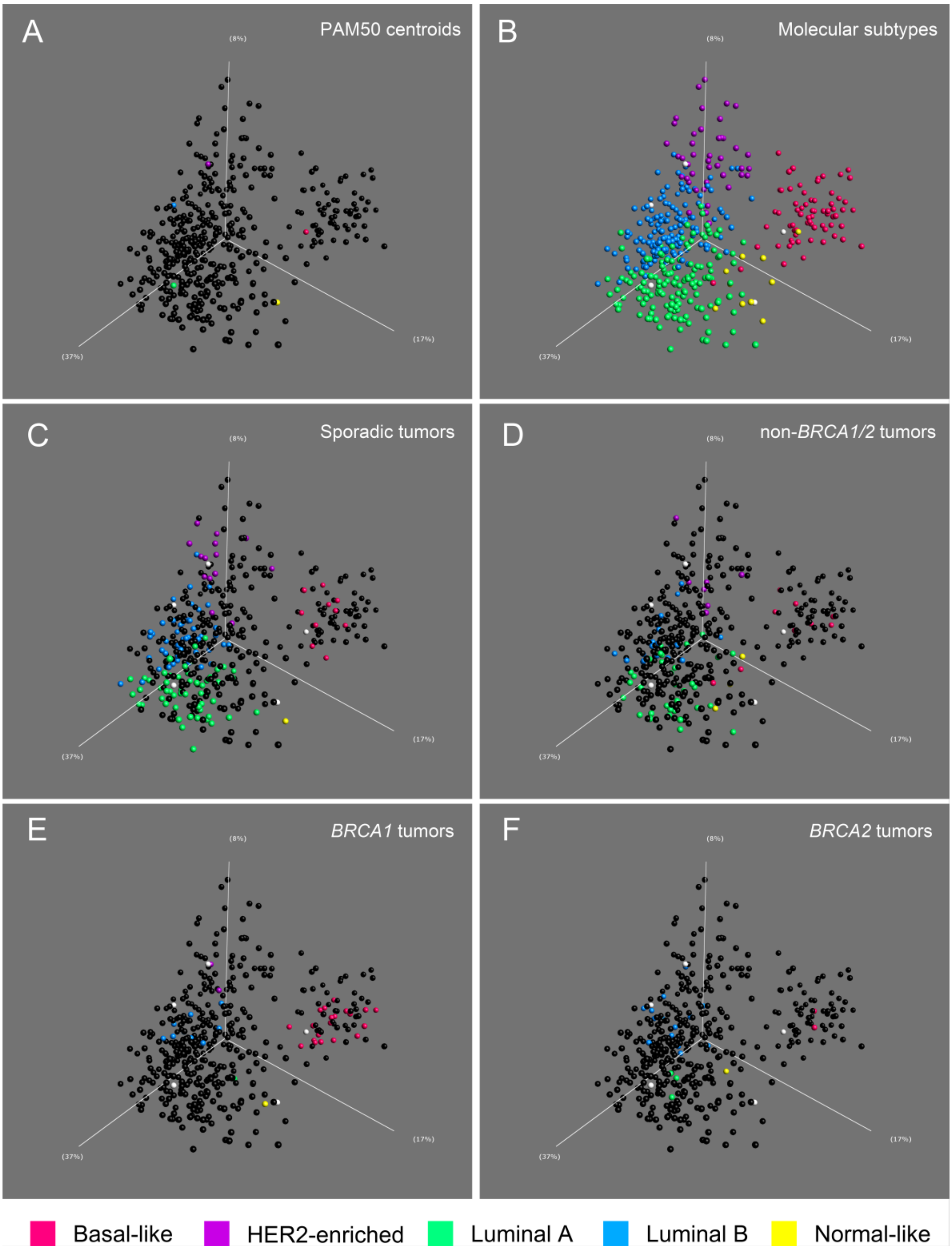

Supplement: Additional file 3: Figure S2. — PCA plots visualizing molecular subtype classifications. [file 1755-8794-7-9-S3.pdf]
