# Supplementary material for: RNA profiling reveals familial aggregation of molecular subtypes in non-BRCA1/2 breast cancer families
Source: BMC Med Genomics. 2014 Jan 31;7:9. doi: 10.1186/1755-8794-7-9 (PMC3909442; doi:10.1186/1755-8794-7-9)
Supplement: Additional file 4: Table S1. — Distribution of predicted molecular subtypes within BRCA1, BRCA2, non-BRCA1/2 and sporadic tumors using PAM50 signature. [file 1755-8794-7-9-S4.pdf]

Table S1. Distribution of predicted molecular subtypes within *BRCA1*, *BRCA2*, non-*BRCA1/2* and sporadic tumors using PAM50 signature.

	**Basal-like**	**HER2-enriched**	**Luminal A**	**Luminal B**	**Normal-like**
**non-*BRCA1/2***	9 (13%)	7 (10%)	33 (47%)	18 (26%)	3 (4%)
BRCA1	20 (61%)	2 (6%)	1 (3%)	9 (27%)	1 (3%)
BRCA2	2 (9%)	0 (0%)	3 (14%)	16 (73%)	1 (4%)
**Sporadic**	10 (8%)	14 (11%)	55 (43%)	48 (37%)	1 (1%)
