# Supplementary material for: RNA profiling reveals familial aggregation of molecular subtypes in non-BRCA1/2 breast cancer families
Source: BMC Med Genomics. 2014 Jan 31;7:9. doi: 10.1186/1755-8794-7-9 (PMC3909442; doi:10.1186/1755-8794-7-9)
Supplement: Additional file 5: Table S2. — Patients characteristics and BRCA1-like and BRCA2-like predictions results for familial non-BRCA1/2 patients. [file 1755-8794-7-9-S5.pdf]

Table S2. Patients characteristics and *BRCA1*-like and *BRCA2*-like predictions results for familial non-*BRCA1/2* patients.

SampleID	FamilyID	Age	Subtype	Type	Grade	*BRCA1*-like prediction	*BRCA2*-like prediction	*BRCA1* promoter methyaltion
A068	008	32	Basal	IDC	3	Sporadic-like		
A037	031	37	Basal	IDC	3	*BRCA1*-like		x
A164	026	39	Basal	IDC	3	*BRCA1*-like		
A154	023	42	Basal	IDC	2	*BRCA1*-like		
A083	031	45	Basal	IDC	3	*BRCA1*-like		x
A176	001	47	Basal	Other	NA	Sporadic-like		
A049	051	47	Basal	Medullary	NA	*BRCA1*-like		X
A064	027	47	Basal	IDC	3	*BRCA1*-like		
A151	035	53	Basal	IDC	3	*BRCA1*-like		
A078	047	29	Her2	IDC	3			
A099	018	29	Her2	IDC	3			
A022	028	36	Her2	IDC	2			
A060	022	61	Her2	IDC	3			
A139	055	65	Her2	IDC	2			
A113	056	70	Her2	IDC	3			
A046	018	81	Her2	IDC	NA			
A066	037	34	LumA	ILC	2			
A076	045	37	LumA	IDC	1			
A062	039	38	LumA	IDC	2			
A077	029	41	LumA	ILC	2			
A056	016	42	LumA	IDC	2			
A157	013	43	LumA	IDC	2			
A026	029	45	LumA	IDC	3			
A034	015	45	LumA	ILC	NA			
A050	003	46	LumA	IDC	1			
A153	017	46	LumA	IDC	2			
A073	032	47	LumA	IDC	2			
A033	005	48	LumA	IDC	2			
A166	010	48	LumA	ILC	2			
A082	019	50	LumA	ILC	3			
A116	058	50	LumA	IDC	2			
A057	020	51	LumA	IDC	1			
A155	002	52	LumA	IDC	2			
A054	038	54	LumA	ILC	NA			
A093	004	55	LumA	IDC	2			
A085	014	57	LumA	IDC	1			
A127	052	57	LumA	IDC	2			
A067	029	59	LumA	IDC	2			
A036	041	61	LumA	IDC	2			
A025	042	64	LumA	IDC	2			
A161	025	66	LumA	IDC	1			
A168	050	66	LumA	IDC	2			
A075	044	68	LumA	ILC	2			
A058	048	70	LumA	IDC	1			
A162	046	71	LumA	ILC	NA			
A074	042	73	LumA	NA	NA			
A052	017	76	LumA	IDC	1			
A135	021	76	LumA	IDC	1			
A043	006	86	LumA	IDC	3			
A097	024	33	LumB	IDC	2		Sporadic-like	
A137	057	35	LumB	NA	NA		*BRCA2*-like	
A055	034	41	LumB	IDC	1		*BRCA2*-like	
A029	034	45	LumB	IDC	3		*BRCA2*-like	
A035	012	45	LumB	IDC	3		*BRCA2*-like	
A061	030	45	LumB	IDC	3		Sporadic-like	
A072	040	50	LumB	ILC	2		Sporadic-like	
A141	054	50	LumB	NA	NA		*BRCA2*-like	
A090	009	54	LumB	NA	NA		*BRCA2*-like	
A158	024	54	LumB	IDC	2		Sporadic-like	
A021	014	59	LumB	IDC	2		Sporadic-like	
A081	049	60	LumB	IDC	3		*BRCA2*-like	
A044	030	62	LumB	IDC	2		Sporadic-like	
A040	027	63	LumB	IDC	1		Sporadic-like	
A041	036	63	LumB	IDC	3		Sporadic-like	
A132	053	64	LumB	IDC	1		Sporadic-like	
A092	043	72	LumB	IDC	2		Sporadic-like	
A098	033	73	LumB	IDC	1		Sporadic-like	
A094	007	36	Normal	IDC	3			x
A045	011	43	Normal	IDC	3			
A028	006	44	Normal	IDC	2			
