# Supplementary material for: RNA profiling reveals familial aggregation of molecular subtypes in non-BRCA1/2 breast cancer families
Source: BMC Med Genomics. 2014 Jan 31;7:9. doi: 10.1186/1755-8794-7-9 (PMC3909442; doi:10.1186/1755-8794-7-9)

Figure S3. Relations between *BRCA1* gene expression and *BRCA1* promoter methylation and PAM50 molecular subtypes.

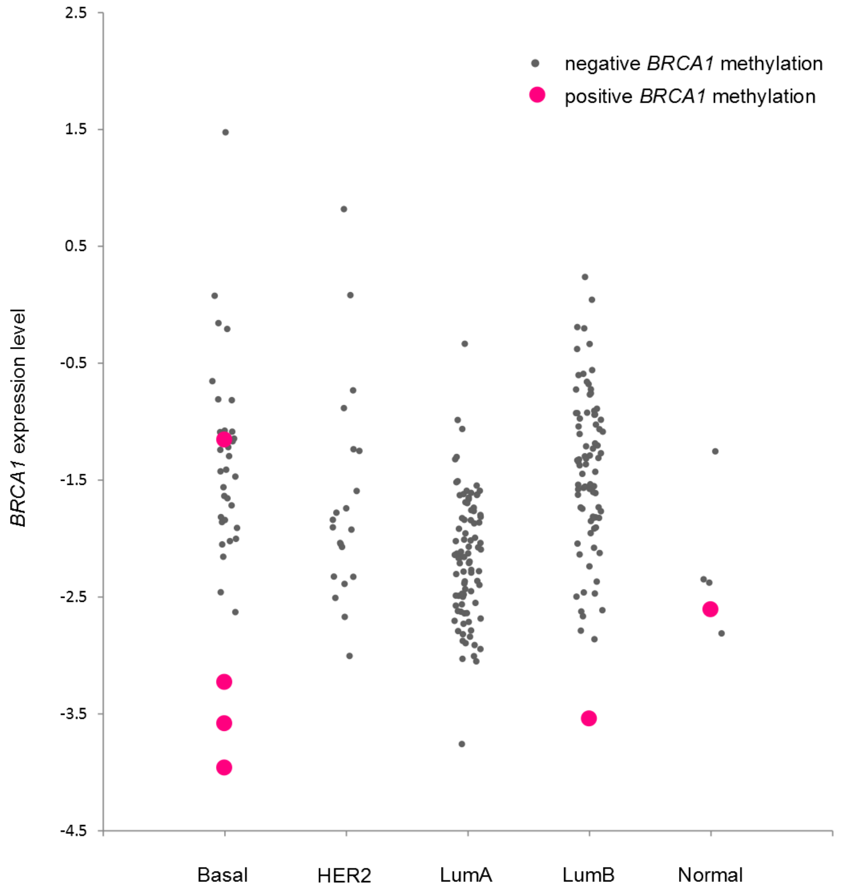

Supplement: Additional file 6: Figure S3. — Relations between BRCA1 gene expression and BRCA1 promoter methylation and PAM50 molecular subtypes. [file 1755-8794-7-9-S6.pdf]
